# Causal relationships between systemic inflammatory cytokines and adhesive capsulitis: a bidirectional Mendelian randomization study

**DOI:** 10.3389/fimmu.2024.1380889

**Published:** 2024-06-24

**Authors:** Yi Ouyang, Miaomiao Dai

**Affiliations:** ^1^ Department of Joint Surgery, Shunde Hospital, Southern Medical University (The First People’s Hospital of Shunde, Foshan), Foshan, Guangdong, China; ^2^ Department of Ophthalmology, Shunde Hospital, Southern Medical University (The First People’s Hospital of Shunde, Foshan), Foshan, Guangdong, China

**Keywords:** systemic inflammatory cytokines, inflammation, adhesive capsulitis, frozen shoulder, Mendelian randomization, causality

## Abstract

**Background:**

Mounting evidence suggests a connection between inflammatory cytokines and adhesive capsulitis (AC). However, the specific systemic inflammatory cytokines contributing to AC have not been clearly identified. This study employed Mendelian randomization (MR) to explore the causal relationships between 41 inflammatory cytokines and AC.

**Methods:**

In this bidirectional, two-sample MR analysis, genetic variations associated with AC were derived from a comprehensive genome-wide association study (GWAS). The inflammatory cytokines data were sourced from a GWAS summary involving 8,293 healthy participants. The primary MR method employed was inverse variance weighting, supplemented by MR-Egger, weighted median, and MR-pleiotropy residual sum and outlier for sensitivity analysis. Heterogeneity was assessed using Cochran’s Q test, and the MR results were validated using the leave-one-out method.

**Results:**

Elevated levels of interferon gamma-induced protein 10 (IP-10) (odds ratio (OR) = 1.086, 95% confidence interval (CI) = 1.002–1.178) and regulated on activation, normal T cell expressed and secreted (RANTES) (OR = 1.107, 95% CI = 1.026–1.195) were linked to an increased risk of AC. Increased levels of stromal cell-derived factor-1 alpha (SDF-1α) (OR = 0.879, 95% CI = 0.793–0.974) and tumor necrosis factor-alpha (TNF-α) (OR = 0.911, 95% CI = 0.831–0.999) were associated with a reduced AC risk. Moreover, genetically predicted AC exhibited associations with elevated cutaneous T cell attracting (CTACK) levels (OR = 1.202, 95% CI = 1.007–1.435) and diminished levels of interleukin-17 (IL-17) (OR = 0.678, 95% CI = 0.518–0.888) and interleukin-5 (IL-5) (OR = 0.786, 95% CI = 0.654–0.944), as confirmed through inverse-variance weighted (IVW) methods.

**Conclusion:**

The present study successfully establishes a causal association between genetically proxied circulating levels of IP-10, RANTES, SDF-1α, and TNF-α and the risk of AC. Additionally, AC contributes to an increase in CTACK and a decrease in IL-17 and IL-5. This significant finding not only enhances the understanding of the pathogenesis of AC but also holds promise for the development of effective clinical management strategies.

## 1 Introduction

Adhesive capsulitis (AC), commonly referred to as “frozen shoulder,” is a prevalent ailment, impacting an estimated 2–5% of the general population (1). The actual incidence may be higher due to the condition being typically mild and self-limited, resulting in numerous cases going unreported and untreated (1). Characterized by a pathological progression involving gradual fibrosis of the glenohumeral joint, AC manifests with constrained active and passive range of motion, joint capsule contracture, and shoulder discomfort (2). Codman’s work in 1934 marked a seminal depiction of AC as a painful constriction of shoulder mobility. Subsequently, the subject area was refined by Neviaser in 1945, who delineated AC as a pain-constrained restriction in glenohumeral range of motion (ROM) lacking structural deficits, thereby coining the term “adhesive capsulitis” (3). Primarily afflicting women aged 40 to 60, initial patient complaints regarding AC include pain during extreme ROM, persisting for at least a month, followed by the onset of joint limitation, notably in flexion, abduction (both at average and extreme angles), and external rotation (particularly between 45 and 90 degrees of abduction), significantly impeding daily activities (4). Though AC is conventionally perceived as self-resolving within 1 to 3 years, lingering symptoms persist in 20–50% of patients (5, 6).

The pathophysiological underpinnings of AC remain unclear, with proposed mechanisms encompassing inflammatory changes, fibrosis, and capsular contracture (7). Associations with diabetes mellitus, hypothyroidism, Dupuytren’s contracture, and breast cancer treatment underscore its correlation with immune system perturbation and heightened inflammatory response (8). Notably, elevated levels of pro-inflammatory cytokines, including interleukin (IL)-1α, IL-1β, IL-6, IL-8, IL-17, and tumor necrosis factor-alpha (TNF-α), is a feature prominent in individuals with AC that fosters a pro-inflammatory milieu (9–11). Nevertheless, there exists debate regarding whether inflammatory cytokines are causative agents or consequential to disease progression and medication use following AC onset. Observational studies addressing this conundrum may be confounded by unforeseen variables or reverse causation, precluding definitive causal correlations.

Mendelian randomization (MR) is an analytical paradigm for discerning causal relationships between exposure and outcome through genetic variations in non-experimental data (12). Accounting for the random allele assignment during meiosis, MR effectively mitigates conventional confounding variables and reverse causation, thereby bolstering the evidentiary basis for causal inference (13). Leveraging two-sample MR analysis, researchers can scrutinize instrument–exposure and instrument–outcome relationships across distinct population samples, augmenting the versatility and efficacy of the analytical approach (14). This study utilized valid genetic variants from published genome-wide association study (GWAS) summary data encompassing 41 inflammatory cytokines to scrutinize their associations with AC, subsequently probing the direction of causation through the inversion of exposure and outcome variables.

## 2 Materials and methods

### 2.1 Study design

**Figure 1:**
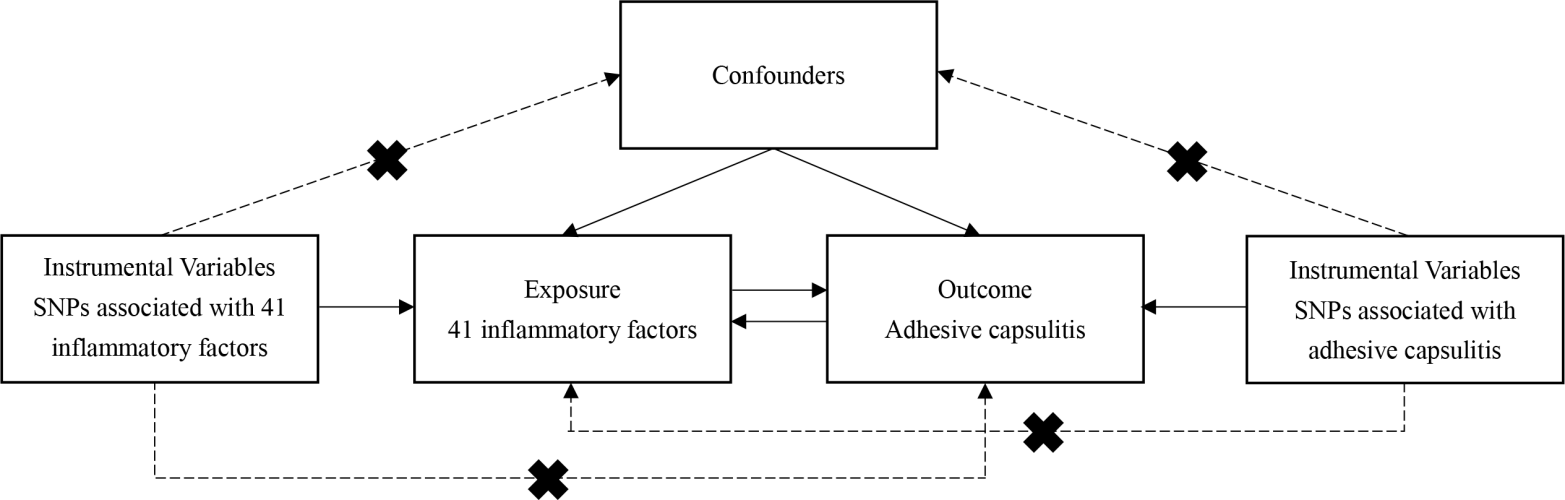
Flowchart of the study. The three assumptions of the MR study. Assumption 1: The instrumental variable, chosen as the genetic variation, must genuinely correlate with the targeted exposure. Assumption 2: The selected genetic variation should remain unrelated to any confounding factors. Assumption 3: The genetic variation must solely influence the outcome through the designated exposure.

The bidirectional MR analysis, depicted in **Figure 1**, forms the crux of this study’s investigative framework. This analytical approach relies on three pivotal assumptions: 1) the instrumental variable (IV), chosen as the genetic variation, must genuinely correlate with the targeted exposure; 2) the selected genetic variation should remain unrelated to any confounding factors; and 3) the genetic variation must solely influence the outcome through the designated exposure (15).

The study utilized two distinct sets of GWAS databases encompassing 41 systemic inflammatory cytokines and AC and unfolds in two phases. Firstly, the causal interplay between inflammatory cytokines and AC was ascertained by employing genetic variations associated with each inflammatory factor. Subsequently, the genetic variations linked to AC were explored to delineate the reciprocal causal relationship with inflammatory cytokines. Notably, the exclusive derivation of all GWAS data from individuals of European ancestry constituted a secondary analysis of previously published data, eliminating the need for additional ethical approvals.

### 2.2 Data sources

This study utilized the extensive GWAS meta-analysis of circulating concentrations of 41 cytokines, comprising data from 8,293 Finnish individuals across three independent population cohorts: the Cardiovascular Risk in Young Finns Study, FINRISK1997, and FINRISK2002 (16). AC data, sourced from a combined GWAS analysis of FinnGen and the UK Biobank, integrates information from 10,104 cases identified through inpatient, surgical, and primary care codes, thereby constituting the most comprehensive GWAS data on AC involving individuals of European ancestry (**Supplementary Table S1**) (17).

### 2.3 Selection of IVs

To align with the stringent MR assumptions illustrated in **Figure 1**, the study considered all single nucleotide polymorphisms (SNPs) predicting exposures at genome-wide significance (*P* < 5 × 10^-8^) to exhibit strong and independent prediction (r^2^ < 0.001 within 10 Mb). As only eight systemic inflammatory cytokines had three or more independent SNPs reaching genome-wide significance, the study adopted a less stringent threshold of 5 × 10^-6^ to enhance SNP availability for inflammatory cytokines. The thresholds were used to select genetic independent variables, as described before (18). Following these steps, a total of 41 distinct types of inflammatory cytokines were identified. SNPs with F-statistics less than 10, indicating weak IVs, were excluded. In adherence to MR principles, target SNPs were screened to eliminate those associated with the results. The effect alleles of the genetic variants were meticulously coordinated in both the exposure and outcome GWAS, **Supplementary Tables S2** and **S3**.

### 2.4 Data analysis

The inverse-variance weighted (IVW) method was employed as the primary approach to estimate the causal effect of exposure on the outcome, adhering to the fundamental principles of an MR study for precise causal estimation. Additional complementary methods, such as the weighted median (WM) method and MR-Egger regression, were also utilized in diverse scenarios (19). The WM method, which utilizes the median MR estimate as the causal estimate, offers benefits over MR-Egger regression by reducing type I error and enhancing the power of causal estimation. MR-Egger regression incorporates the reciprocal of resultant variances as weights for the analysis and differs from the IVW method by considering the presence of an intercept term in the regression analysis. The intercept in the MR-Egger regression model enables detecting horizontal pleiotropy, whereby a *P*-value < 0.05 is considered statistically significant (20). Horizontal pleiotropy indicates that genetic IVs independently influence outcomes, which contradicts the definition of IVs. Sensitivity analyses, as presented in **Table 1**, further ensured the robustness of the findings. The Cochran’s Q test, also detailed in **Table 1**, was employed to assess heterogeneity between SNPs. In instances where heterogeneity was present (*P* < 0.05), certain SNPs with small *P*-values were omitted, or a random-effects model was directly utilized to evaluate the MR effect. Finally, a leave-one-out analysis, depicted in **Supplementary Figures S1**-**S7**, was conducted to assess the stability of the results. The TwoSample package (21) and MR-PRESSO (22) in R (version 4.3.1) were employed for the analysis.

**Table 1:** Heterogeneity test of the IVW and MR egger analyses and pleiotropy test (egger intercept).

Exposure	Outcome	Methods	Cochran’s Q	Q-value	*P*-value (Pleiotropy test)
interferon gamma-induced protein 10 (IP10)	adhesive capsulitis	MR egger	4.386	0.495	0.683
		Inverse variance weighted	4.574	0.600	
regulated on activation, normal T cell expressed and secreted (RANTES)	adhesive capsulitis	MR egger	1.781	0.939	0.474
		Inverse variance weighted	2.363	0.937	
stromal cell-derived factor-1 alpha (SDF1α)	adhesive capsulitis	MR egger	1.775	0.777	0.729
		Inverse variance weighted	1.913	0.861	
tumor necrosis factor-alpha (TNF-α)	adhesive capsulitis	MR egger	1.394	0.498	0.474
		Inverse variance weighted	2.159	0.540	
adhesive capsulitis	Cutaneous T-cell attracting (CTACK)	MR egger	15.014	0.594	0.783
		Inverse variance weighted	15.092	0.656	
adhesive capsulitis	interleukin-17 (IL-17)	MR egger	0.060	0.970	0.338
		Inverse variance weighted	1.617	0.656	
adhesive capsulitis	IL-5	MR egger	13.480	0.704	0.880
		Inverse variance weighted	13.503	0.761	

## 3 Results

The study selected 362 SNPs as IVs for 41 systemic inflammatory regulators, adhering to predefined guidelines to guarantee the suitability of the chosen SNPs. Notably, the F-statistics for each SNP utilized in the analysis surpassed 10, underscoring the robust nature of the IVs. As a result, no weak biases were observed in the outcomes, solidifying the reliability of the conclusions drawn from this study.

**Figure 2:**
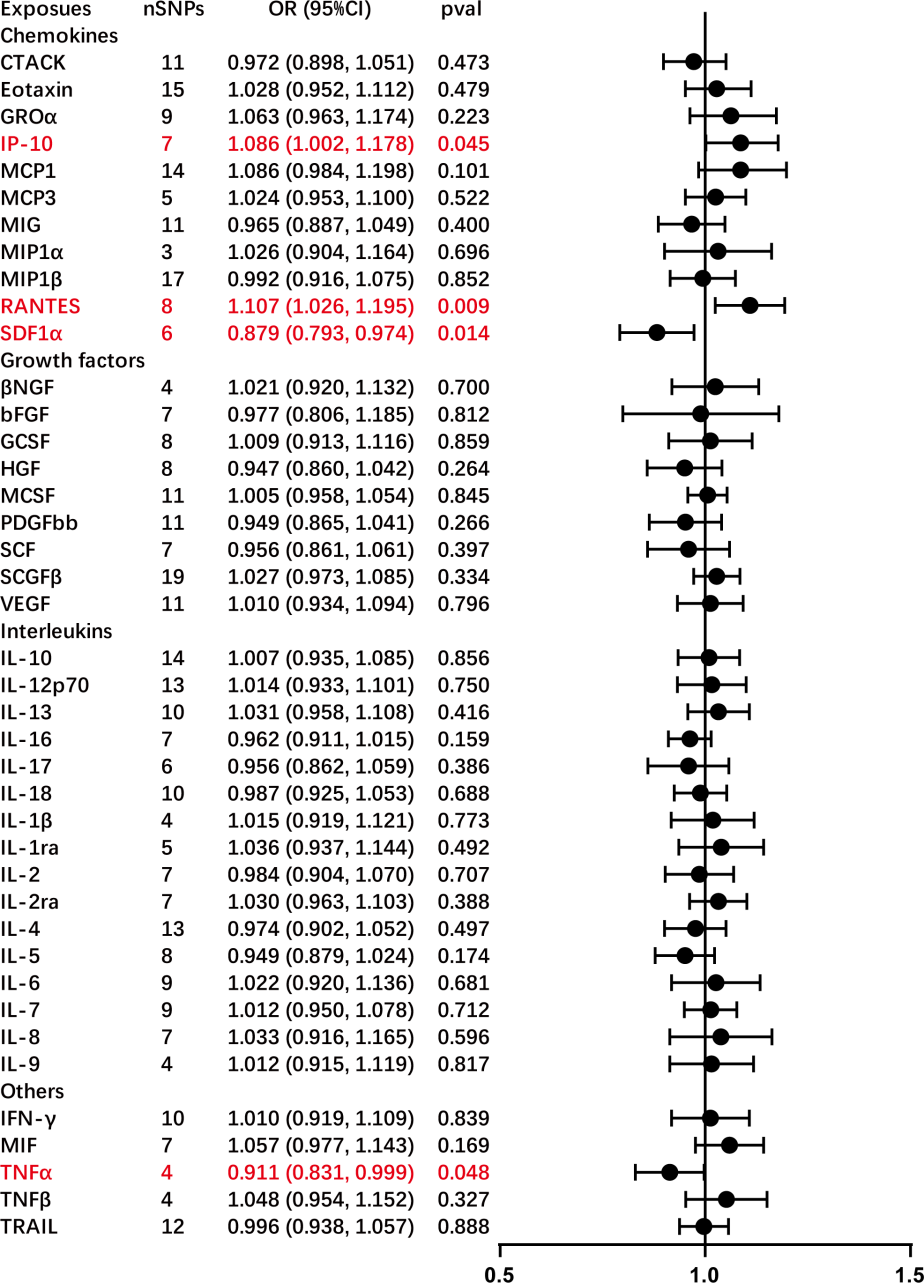
Causal correlations of 41 inflammatory cytokines on adhesive capsulitis (AC). The change in the odds ratio (OR) of AC per one standard deviation (SD) rise in the cytokine level is shown by OR and 95% confidence interval. The results from the inverse variance weighted method are shown for all cytokines. bNGF, beta nerve growth factor; CTACK, cutaneous T cell-attracting chemokine; FGFBasic, basic fibroblast growth factor; GCSF, granulocyte colony-stimulating factor; GROa, growth-regulated oncogene-a; HGF, hepatocyte growth factor; IFNg, interferon gamma; IL, interleukin; IP-10, interferon gamma-induced protein 10; MCP1, monocyte chemotactic protein 1; MCP3, monocyte-specific chemokine 3; MCSF, macrophage colony-stimulating factor; MIF, macrophage migration inhibitory factor; MIG, monokine induced by interferon gamma; MIP1a, macrophage inflammatory protein-1a; MIP1b, macrophage inflammatory protein-1b; PDGFbb, platelet-derived growth factor bb; RANTES, regulated upon activation normal T cell expressed and secreted factor; SCF, stem cell factor; SCGFb, stem cell growth factor beta; SDF-1a, stromal cell-derived factor-1 alpha; SNPs, single-nucleotide polymorphisms; TNF-a, tumor necrosis factor alpha; TNF-b, tumor necrosis factor beta; TRAIL, TNF-related apoptosis-inducing ligand; VEGF, vascular endothelial growth factor.

The primary outcomes from the principal MR analyses of the 41 cytokines are visually depicted in **Figure 2**, with detailed findings available in **Supplementary Table S2**. The genetically predicted systemic inflammatory cytokines exhibited evident associations with AC, as corroborated by the subsequent outcomes. Elevated levels of interferon gamma-induced protein 10 (IP-10) (odds ratio (OR) = 1.086, 95% confidence interval (CI) = 1.002–1.178, *P* = 0.045) and regulated on activation, normal T cell expressed and secreted (RANTES) (OR = 1.107, 95% CI = 1.026–1.195, *P* = 0.009) were linked to an increased risk of AC, as ascertained through the IVW methods (**Table 2**). The MR-Egger intercept failed to identify potential horizontal pleiotropy (*P*-value > 0.05). Additionally, MR-Egger and IVW heterogeneity tests revealed an absence of conspicuous heterogeneity (*P*-value > 0.05). Sensitivity analyses via leave-one-out investigations revealed negligible influence (**Supplementary Figures S1**, **S2**). Notably, the inverse relationship, where higher levels of stromal cell-derived factor-1 alpha (SDF-1α) (OR = 0.879, 95% CI = 0.793–0.974, *P* = 0.014) and TNF-α (OR = 0.911, 95% CI = 0.831–0.999, *P* = 0.048) were associated with a reduced AC risk, as determined via IVW methods, with no indications of heterogeneity or horizontal pleiotropy in the results (*P*-value > 0.05). Comprehensive details are tabulated in **Tables 1** and **2** and **Supplementary Figures S3** and **S4**.

**Table 2:** Bidirectional Mendelian randomization estimates of cytokines and meningiomas (IVW, MR-egger, weighted median, MR-PRESSO).

Exposure	Outcome	Number of SNPs	Methods	OR (95% CI)	*P*-value
interferon gamma-induced protein 10 (IP10)	adhesive capsulitis	7	MR egger	1.058 (0.915, 1.223)	0.482
			Weighted median	1.077 (0.969, 1.198)	0.169
			Inverse variance weighted	1.086 (1.002, 1.178)	0.045
regulated on activation, normal T cell expressed and secreted (RANTES)	adhesive capsulitis	8	MR egger	1.188 (0.976, 1.447)	0.137
			Weighted median	1.154 (1.043, 1.277)	0.006
			Inverse variance weighted	1.107 (1.026, 1.195)	0.009
stromal cell-derived factor-1 alpha (SDF1α)	adhesive capsulitis	6	MR egger	0.858 (0.729, 1.010)	0.139
			Weighted median	0.860 (0.755, 0.979)	0.023
			Inverse variance weighted	0.879 (0.793, 0.974)	0.014
tumor necrosis factor-alpha (TNF-α)	adhesive capsulitis	4	MR egger	0.848 (0.704, 1.021)	0.224
			Weighted median	0.896 (0.802, 1.000)	0.050
			Inverse variance weighted	0.911 (0.831, 0.999)	0.048
adhesive capsulitis	Cutaneous T-cell attracting (CTACK)	19	MR egger	1.298 (0.735, 2.295)	0.381
			Weighted median	1.162 (0.912, 1.480)	0.223
			Inverse variance weighted	1.202 (1.007, 1.435)	0.042
adhesive capsulitis	interleukin-17 (IL-17)	4	MR egger	4.935 (0.216, 112.719)	0.423
			Weighted median	0.719 (0.514, 1.006)	0.054
			Inverse variance weighted	0.678 (0.518, 0.888)	0.005
adhesive capsulitis	IL-5	19	MR egger	0.752 (0.417, 1.356)	0.356
			Weighted median	0.898 (0.699, 1.154)	0.401
			Inverse variance weighted	0.786 (0.654, 0.944)	0.010

**Figure 3:**
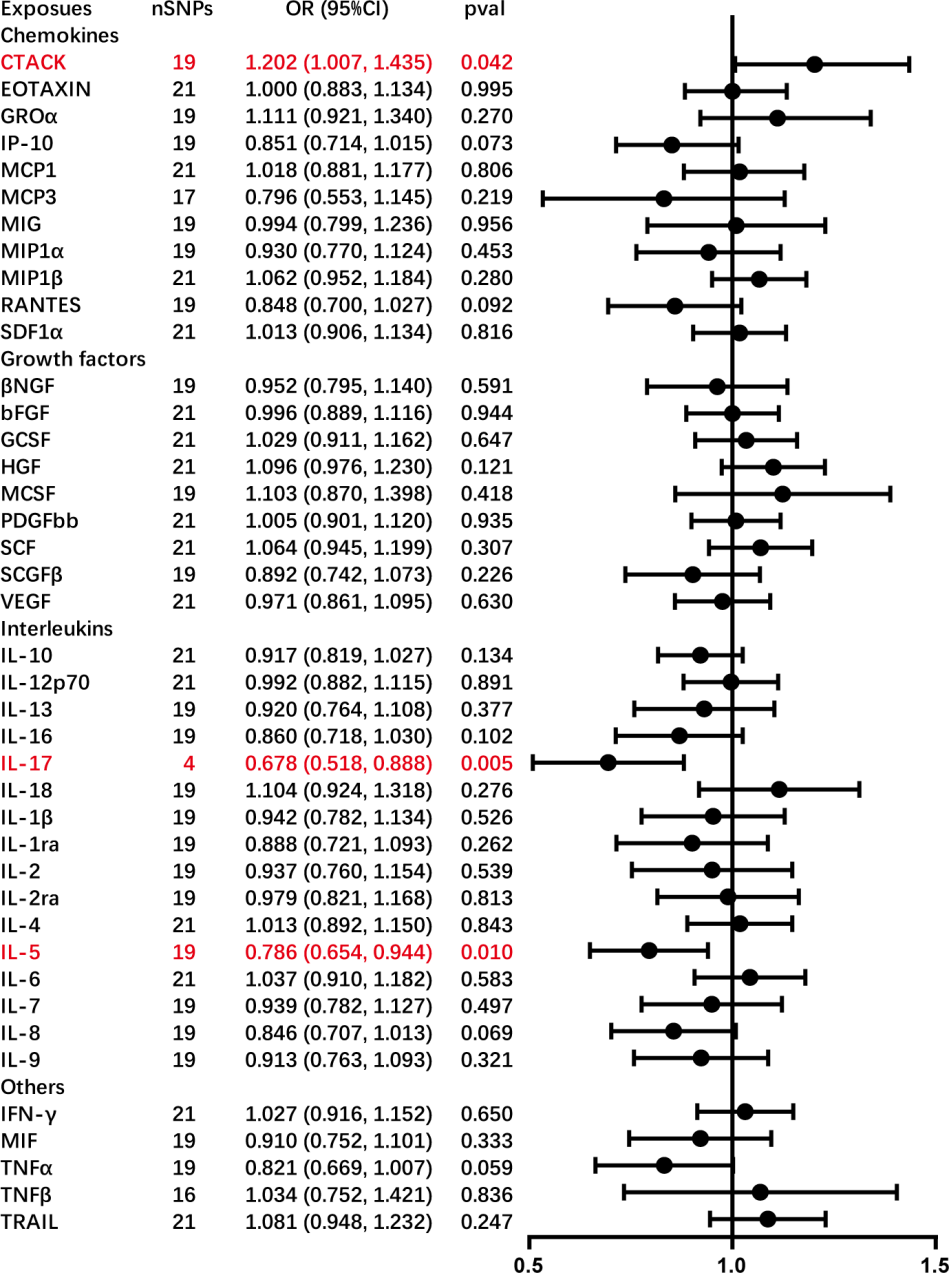
Causal correlations of adhesive capsulitis (AC) on 41 inflammatory cytokines. The change in the standard deviation (SD) of inflammatory cytokines per log odds increase in AC is represented by beta and the 95% confidence interval. The results from the inverse variance weighted method are shown for all cytokines. bNGF, beta nerve growth factor; CTACK, cutaneous T cell-attracting chemokine; FGFBasic, basic fibroblast growth factor; GCSF, granulocyte colony-stimulating factor; GROa; growth-regulated oncogene-a; HGF, hepatocyte growth factor; IFNg, interferon gamma; IL, interleukin; IP-10; interferon gamma-induced protein 10; MCP1, monocyte chemotactic protein 1; MCP3, monocyte-specific chemokine 3; MCSF, macrophage colony-stimulating factor; MIF, macrophage migration inhibitory factor; MIG, monokine induced by interferon gamma; MIP1a, macrophage inflammatory protein-1a; MIP1b, macrophage inflammatory protein-1b; PDGFbb, platelet-derived growth factor bb; RANTES, regulated upon activation normal T cell expressed and secreted factor; SCF, stem cell factor; SCGFb, stem cell growth factor beta; SDF-1a, stromal cell-derived factor-1 alpha; SNPs, single-nucleotide polymorphisms; TNF-a, tumor necrosis factor alpha; TNF-b, tumor necrosis factor beta; TRAIL, TNF-related apoptosis-inducing ligand; VEGF, vascular endothelial growth factor.

This study observed an inherent association between genetically predicted AC and cytokine levels. Significant results from the main MR analyses for AC are depicted in **Figure 3**, with detailed findings in **Supplementary Table S3**. Genetically predicted AC has been found to exhibit associations with elevated levels of cutaneous T cell attracting (CTACK) (OR = 1.202, 95% CI = 1.007–1.435, *P* = 0.042) and diminished levels of IL-17 (OR = 0.678, 95% CI = 0.518–0.888, *P* = 0.005) and IL-5 (OR = 0.786, 95% CI = 0.654–0.944, *P* = 0.010), as confirmed through IVW methods. These outcomes exhibit no evidence of pleiotropy or heterogeneity. Comprehensive details are presented in **Tables 1** and **2** and **Supplementary Figures S5**-**S7**. Diagrammatic representations, including leave-one-out analysis, scatter plot, funnel plot, and forest plot, are available in **Supplementary Figures S1**-**S7**.

## 4 Discussion

The present study is a pioneering and expansive MR inquiry, representing the foremost exploration into the genetic causal interplay between systemic inflammatory cytokines and AC, and vice versa. Previous studies predominantly delved into localized inflammation within the capsule or synovium tissues, neglecting the systemic inflammatory response of the shoulder. Observational studies in clinical settings, often marred by confounding factors and reverse causation bias, inherently lead to distorted causal relationships. This study’s findings revealed a positive association between genetically predicted levels of IP-10 and RANTES and the risk of AC, whereas the levels of SDF-1α and TNF-α exhibited a negative association. Additionally, genetic predisposition to AC suggests an increase in CTACK levels and a decrease in IL-17 and IL-5 levels. These robust findings have been validated by sensitivity analyses, underscoring the genetic regulatory nexus between systemic inflammatory cytokines and AC.

Systemic inflammatory cytokines constitute a group of molecules orchestrating diverse roles in inflammation regulation throughout the body. Maintaining equilibrium between pro-inflammatory and anti-inflammatory processes, these regulators ensure effective immune system functioning during infections, injuries, or diseases while preventing excessive tissue damage. Systemic inflammatory cytokines, including cytokines, chemokines, and various growth factors, collectively coordinate immune response processes (23). AC, an intricate and multifactor disorder linked to inflammatory changes, fibrosis, and capsular contracture, involves systemic inflammatory cytokines in its onset and development.

IP-10, also known as chemokine (C-X-C motif) ligand (CXCL) 10, is a 10 kDa secreted polypeptide categorized within the CXC chemokine family (24). This chemokine can activate integrin and orchestrate directed migration in various cell types, including activated T cells, monocytes, and natural killer cells. As a result, IP-10 plays a pivotal role in regulating inflammation at multiple levels (25). Beyond its fundamental functions, IP-10 exhibits additional pro-inflammatory properties, such as inducing molecules like IL-8 and CXCL-5, as well as the up-regulating costimulatory cell surface molecules (CD54, CD80, CD86, etc.) on monocytes (26). Notably, elevated levels of IP-10 have been observed in knee diseases such as osteoarthritis (OA) and rheumatoid arthritis (RA), suggesting a potential association with the influx of inflammatory cells in synovial tissue (27–29). This raises the intriguing possibility that IP-10 may contribute to the onset of AC, an inflammatory shoulder condition related to synovial tissues. However, despite these possible associations, a paucity of relevant studies persists, highlighting the need for further research to explore this potential relationship comprehensively.

RANTES, a member of the cysteine-cysteine (CC) chemokine family that is also referred to as CC ligand 5 (CCL5), exhibits chemotactic properties on inflammatory cells and various other cell types by activating chemokine receptors (30). Similar to IP-10, the levels of RANTES increase in knee diseases like OA and RA, contributing to a pro-inflammatory milieu in these conditions (29, 31, 32). Notably, Norman et al. investigated the relationship between inflammation biomarkers and musculoskeletal pain, revealing no significant association between RANTES and shoulder pain (33). Despite this, direct studies on the relationship between RANTES and AC are limited, warranting further investigation. Additional research is imperative to delineate the precise role of RANTES in the pathophysiology of AC, exploring its potential as an early predictive indicator, preventive target, and therapeutic focus.

SDF-1, also identified as CXCL12, belongs to the CXC chemokine family (34). The upregulated expression of SDF-1 in OA and RA positions it as a potential therapeutic target for degenerative joint diseases (35). Kim et al. reported an elevation of SDF-1 levels in subacromial bursitis, a component of the pathological process in AC, sharing similar histological features and cell types with AC (36, 37). Contrary to prior studies, this study revealed a negative association between SDF-1α levels and the risk of AC. Interestingly, Wang et al. demonstrated that SDF-1 mitigates the nucleotide-binding oligomerization domain, leucine-rich repeat-containing pyrin domain 3 (NLRP3) inflammasome and pyroptosis in OA synoviocytes by activating the adenosine monophosphate-activated protein kinase (AMPK) signaling pathway, suggesting a potential anti-inflammatory role in OA (38). The intricate role of SDF-1 in joint inflammation necessitates further exploration in the context of AC.

TNF-α is a well-recognized pro-inflammatory cytokine and plays a major role in the pathogenesis of immune-mediated inflammation of the joint (9). Lho et al. observed significantly elevated levels of TNF-α in the joint capsules and subacromial bursae of patients with AC (9). However, Nishimoto et al. noted higher TNF-α expression only in the subacromial bursa of patients with AC compared to those with shoulder instability, with no significant differences in the rotator interval and axillary recess (39). Bunker et al. reported only a slight elevation in TNF-α messenger ribonucleic acid (mRNA) expression in patients with AC, lacking statistical significance (40). The study by Schydlowsky et al. demonstrated no effect of subcutaneous TNF-α blockade injections on AC symptoms (41). Despite these disparate findings, this study revealed a negative association between TNF-α levels and AC risk, emphasizing the need for further exploration into the precise role of TNF-α in AC.

CTACK, also called CC chemokine ligand 27, is the cutaneous T cell attracting chemokine, consistently expressed by epidermal keratinocytes. This chemokine binds to chemokine receptor 10 on skin-homing T cells, suggesting a pivotal role in T cell-mediated inflammation. Despite its well-established presence in the skin, limited research has explored the implications of CTACK in joint biology (42). Endres et al. proposed that CTACK expression is elevated in the synovial fluid of patients with RA compared to normal donors. This heightened expression significantly inhibited the migration of progenitors, indicating a potential regulatory role in the inflammatory processes associated with RA (43). Given the shared microenvironment between AC and RA (36), this study revealed an increase in CTACK levels in the context of AC. This novel finding contributes to the ongoing characterization of CTACK’s potential role in AC and underscores the need for further investigation to elucidate its specific involvement in joint-related inflammation.

The IL family represents a group of lymphatic factors pivotal in activating and differentiating immune cells, as well as influencing processes such as proliferation, maturation, migration, and adhesion. These cytokines exhibit both pro-inflammatory and anti-inflammatory properties, with their primary role being the modulation of growth, differentiation, and activation during inflammatory and immune responses (44). Earlier studies have reported elevated expressions of IL-1α, IL-1β, IL-6, and IL-8 in patients with AC (9, 10, 45). However, the present study did not establish a causal relationship between these ILs and AC. IL-5, a homodimer cytokine, is involved in eosinophil differentiation, recruitment, maturation, activation, and degranulation. Its involvement in allergic and inflammatory immune responses is established in various diseases such as asthma, atopic dermatitis, chronic obstructive pulmonary disease, and eosinophilic gastrointestinal diseases (46). Although previous studies have reported higher IL-5 expression in OA and RA (47, 48), conflicting reports have indicated its absence in some patients with OA and RA (49, 50). Interestingly, this study identified a lower level of IL-5 associated with AC, emphasizing the need for further exploration into the role of IL-5 in the context of AC.

IL-17, a cytokine known for mediating inflammation, fibrosis, and pain signaling, serves as the signature cytokine of the Th17 T-helper cell population (51–54). Akbar et al. demonstrated that IL-17A exhibited significantly greater expression in AC tissue compared to control (11). However, this study found an association between AC and a lower level of IL-17. The limited literature on the role of IL-17 in AC underscores the necessity for additional studies to comprehensively understand its involvement in this condition.

The present study exhibits several strengths. Firstly, it marks the pioneering application of MR to assess the causal relationship between systemic inflammatory cytokines and AC using the latest summary-level data. Many previous investigations into the association between systemic inflammatory cytokines and AC relied on cross-sectional studies and animal models, limiting the ability to establish causality. This bidirectional MR study successfully circumvented reverse causality and mitigated residual confounding. Secondly, this analysis leveraged summary data from the most extensive GWAS meta-analysis on systemic inflammatory cytokines, combined with phenome-wide association study summary data from FinnGen, ensuring the robustness of the instruments for the MR analysis. MR-PRESSO and MR-Egger tests were employed to detect and address horizontal pleiotropy. Thirdly, from a clinical perspective, the study focused on serum, one of the most accessible and easily obtained biofluids, allowing for straightforward sample collection from both patients with AC and healthy controls. This offers an alternative to the highly invasive procedures required for collecting capsule and synovium samples from patients with AC. Furthermore, this study differs from previous research by examining both upstream and downstream circulating biomarkers, offering insights into prediction or treatment strategies for AC.

Nevertheless, it is crucial to acknowledge certain limitations in this study. Firstly, the genetic data predominantly originated from individuals of European descent, potentially limiting the applicability of the findings to other ethnic groups. Caution should be exercised when generalizing the results to diverse populations. Secondly, despite rigorous efforts to exclude SNPs associated with potential confounders and conducting various sensitivity analyses under different assumptions, there still exists a possibility of complex and multidirectional effects not being fully captured. Lastly, though MR analysis is a robust method for estimating causality, it should not replace randomized controlled trials (RCTs). Therefore, the causality inferred from this study may not necessarily align with the results observed in RCTs. It is imperative to conduct individual-based genetic observations and potentially incorporate RCTs in future research to further validate the causal relationships identified here.

## 5 Conclusion

The present study successfully establishes a causal association between genetically proxied circulating levels of IP-10, RANTES, SDF-1α, and TNF-α and the risk of AC. Additionally, AC was found to increase the levels of CTACK and decrease the levels of IL-17 and IL-5. This significant finding not only enhances the understanding of the pathogenesis of AC but also holds promise for developing effective clinical management strategies. Consequently, IP-10, RANTES, SDF-1α, and TNF-α emerge as potential therapeutic targets for the prevention and treatment of AC.

## Data availability statement

The original contributions presented in the study are included in the article/**Supplementary Material**. Further inquiries can be directed to the corresponding author.

## Author contributions

YO: Writing – original draft, Visualization, Validation, Software, Methodology, Investigation, Formal analysis, Data curation, Conceptualization. MD: Writing – review & editing, Supervision, Resources, Project administration.
