# Supplementary material for: Causal relationships between systemic inflammatory cytokines and adhesive capsulitis: a bidirectional Mendelian randomization study
Source: Front Immunol. 2024 Jun 24;15:1380889. doi: 10.3389/fimmu.2024.1380889 (PMC11228239; doi:10.3389/fimmu.2024.1380889)
Supplement: Supplementary file 4 [file Table_4.docx]

Supplementary Material

Causal relationships between systemic inflammatory cytokines and adhesive capsulitis: a bidirectional Mendelian randomization study

Yi Ouyang ^1^, Miaomiao Dai ^2*^

^1^Department of Joint Surgery, Shunde Hospital, Southern Medical University (The First People's Hospital of Shunde, Foshan), No.1 Jiazi Road, Lunjiao, Shunde District, Foshan City, Guangdong Province, China

^2^Department of Ophthalmology, Shunde Hospital, Southern Medical University (The First People's Hospital of Shunde, Foshan), No.1 Jiazi Road, Lunjiao, Shunde District, Foshan City, Guangdong Province, China

*** Correspondence:**Miaomiao Dai
dmm772665671@163.com

## Supplementary Figures


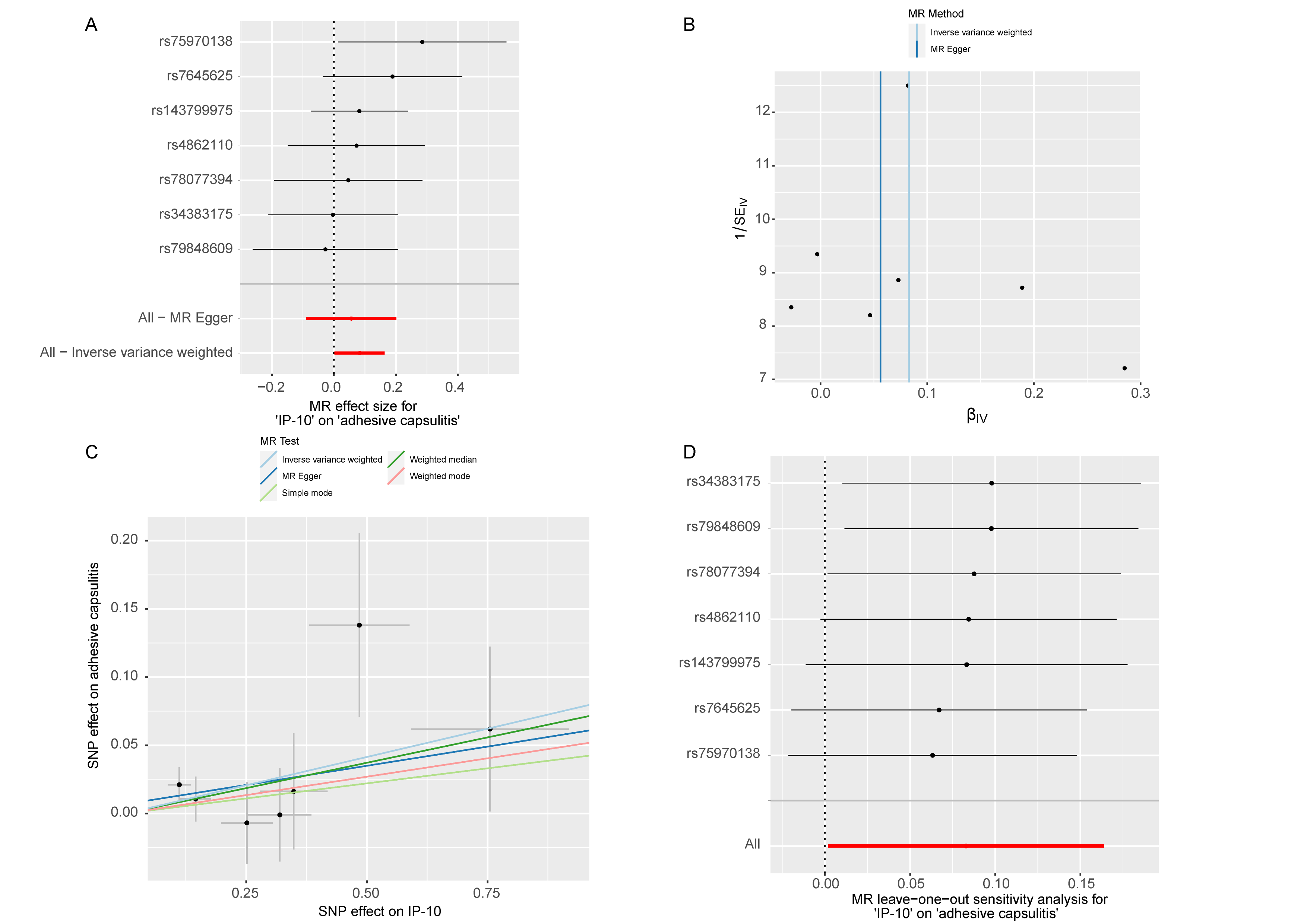


**Supplementary Figure 1.** Forest Plot, Funnel Plot, Scatter Plot, and Leave-one-out Analysis (A-D) of IP-10 on adhesive capsulitis.


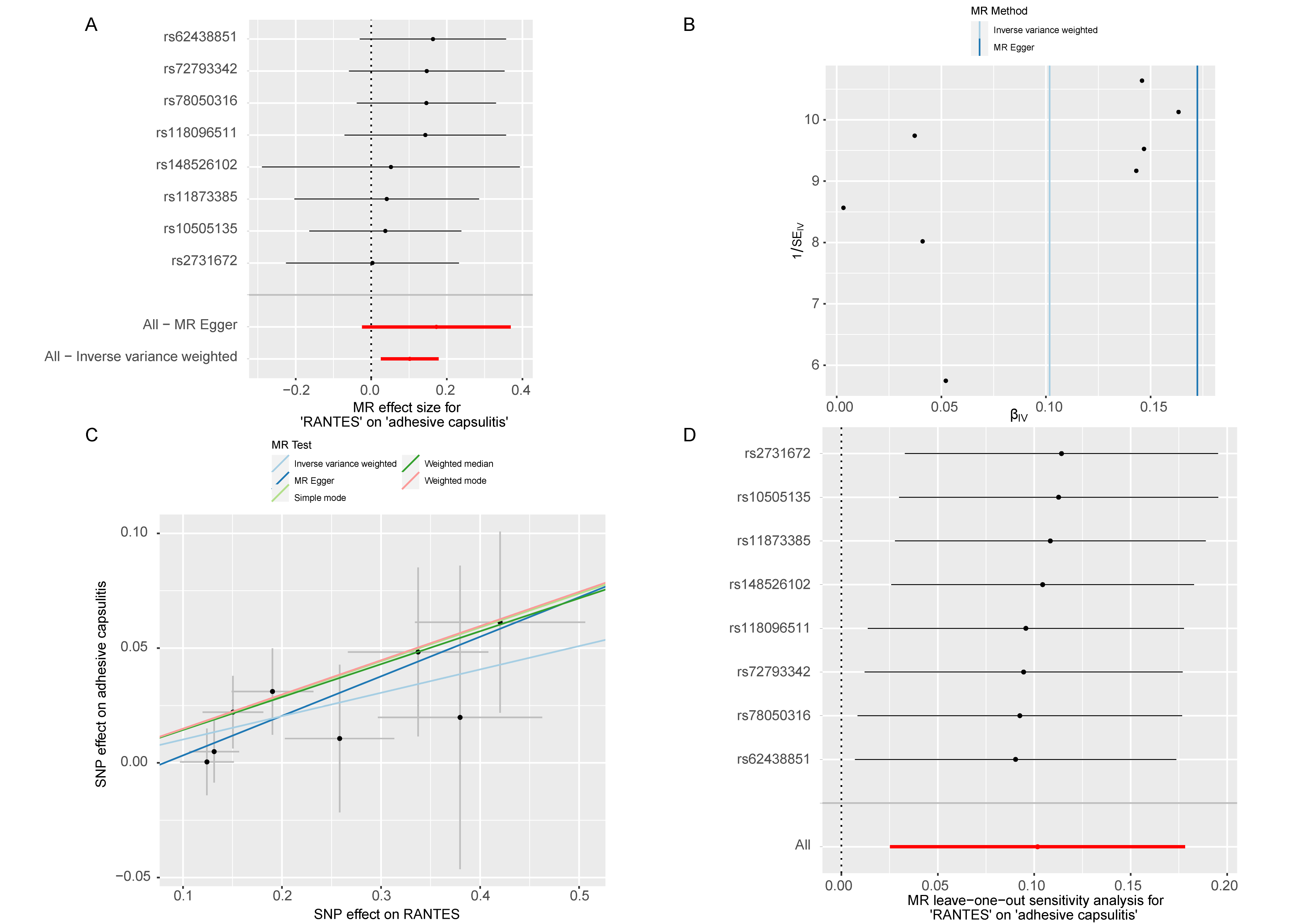


**Supplementary Figure 2** Forest Plot, Funnel Plot, Scatter Plot, and Leave-one-out Analysis (A-D) of RANTES on adhesive capsulitis.


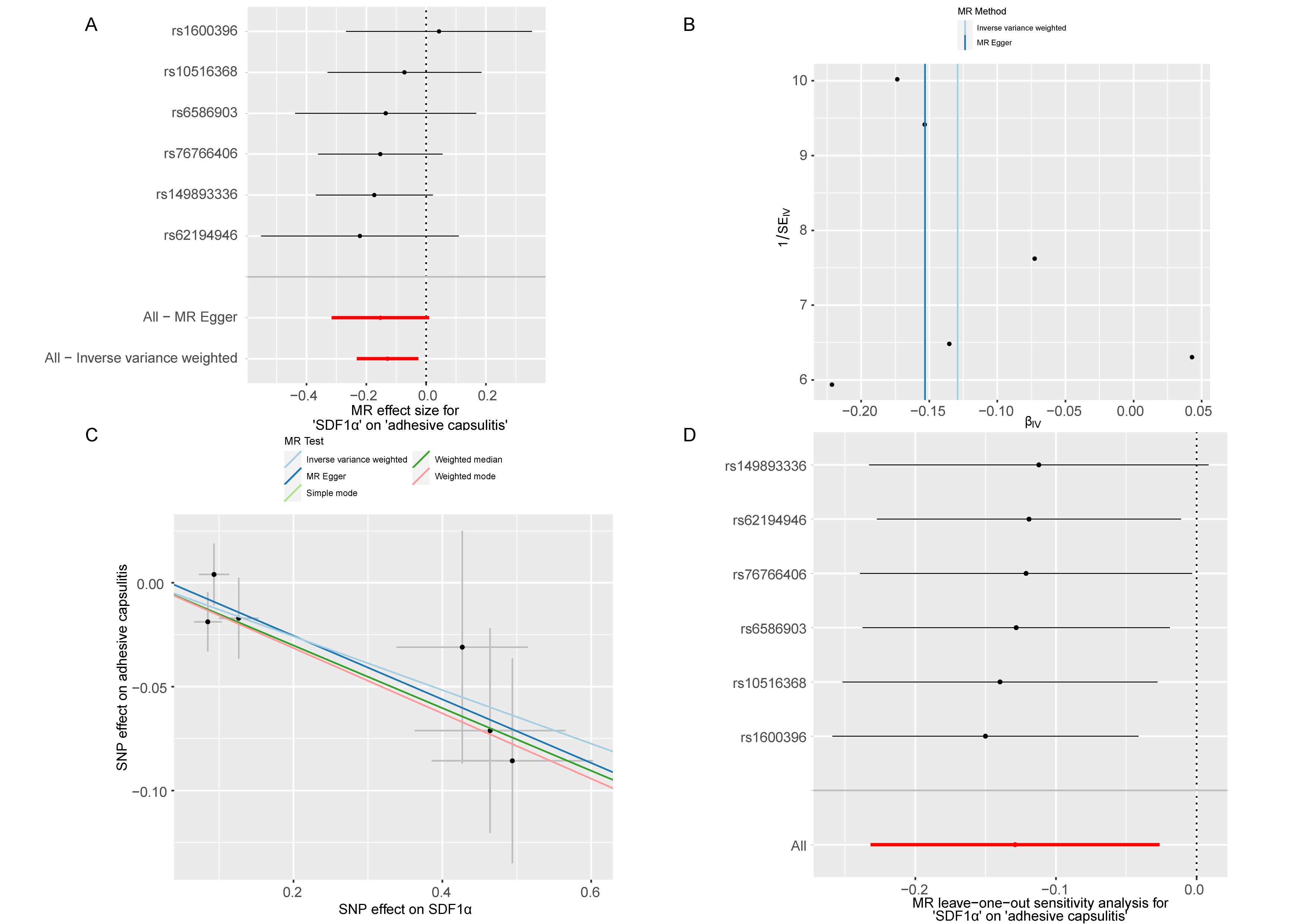


**Supplementary Figure 3** Forest Plot, Funnel Plot, Scatter Plot, and Leave-one-out Analysis (A-D) of SDF1α on adhesive capsulitis.


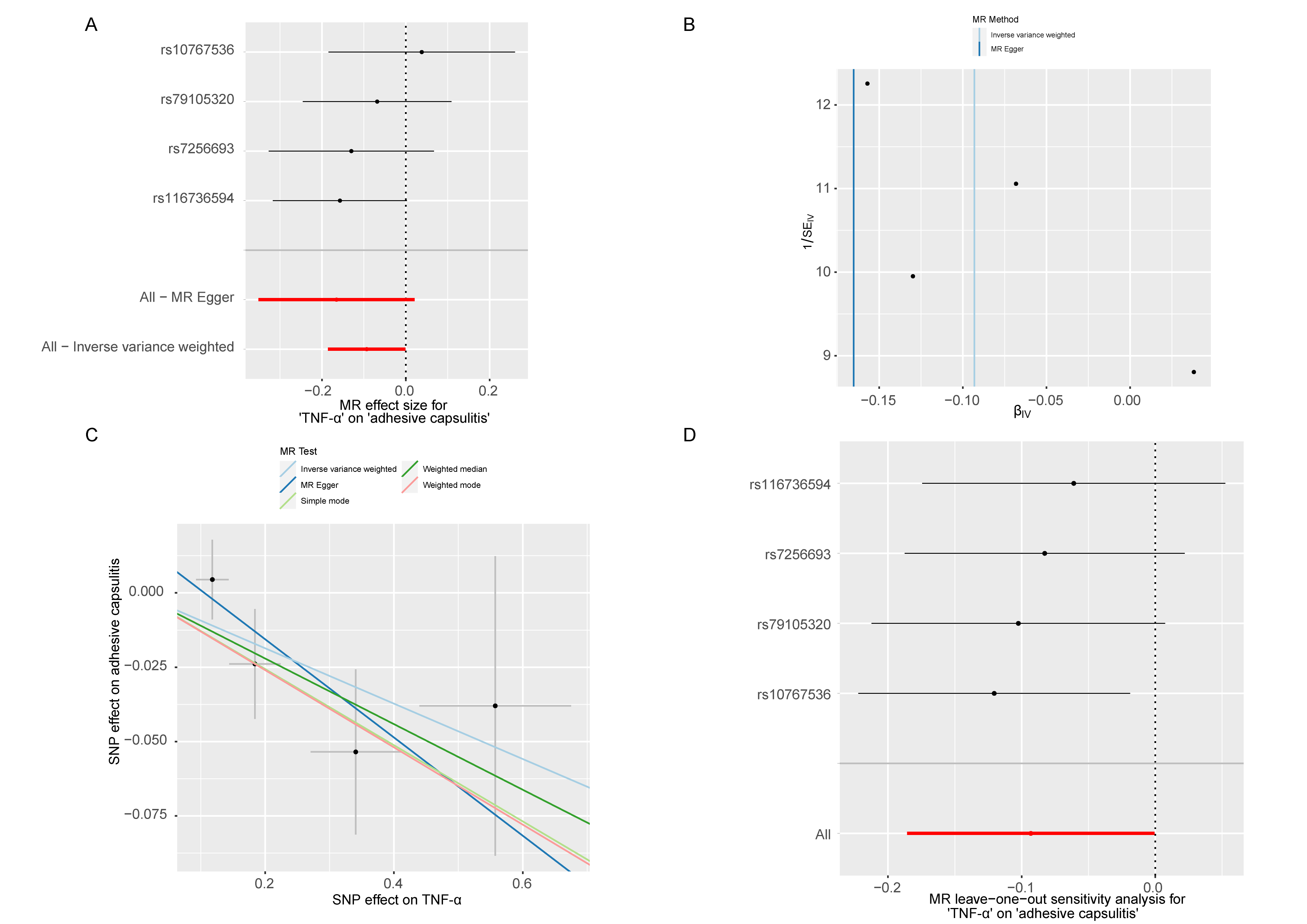


**Supplementary Figure 4** Forest Plot, Funnel Plot, Scatter Plot, and Leave-one-out Analysis (A-D) of TNF-α on adhesive capsulitis.


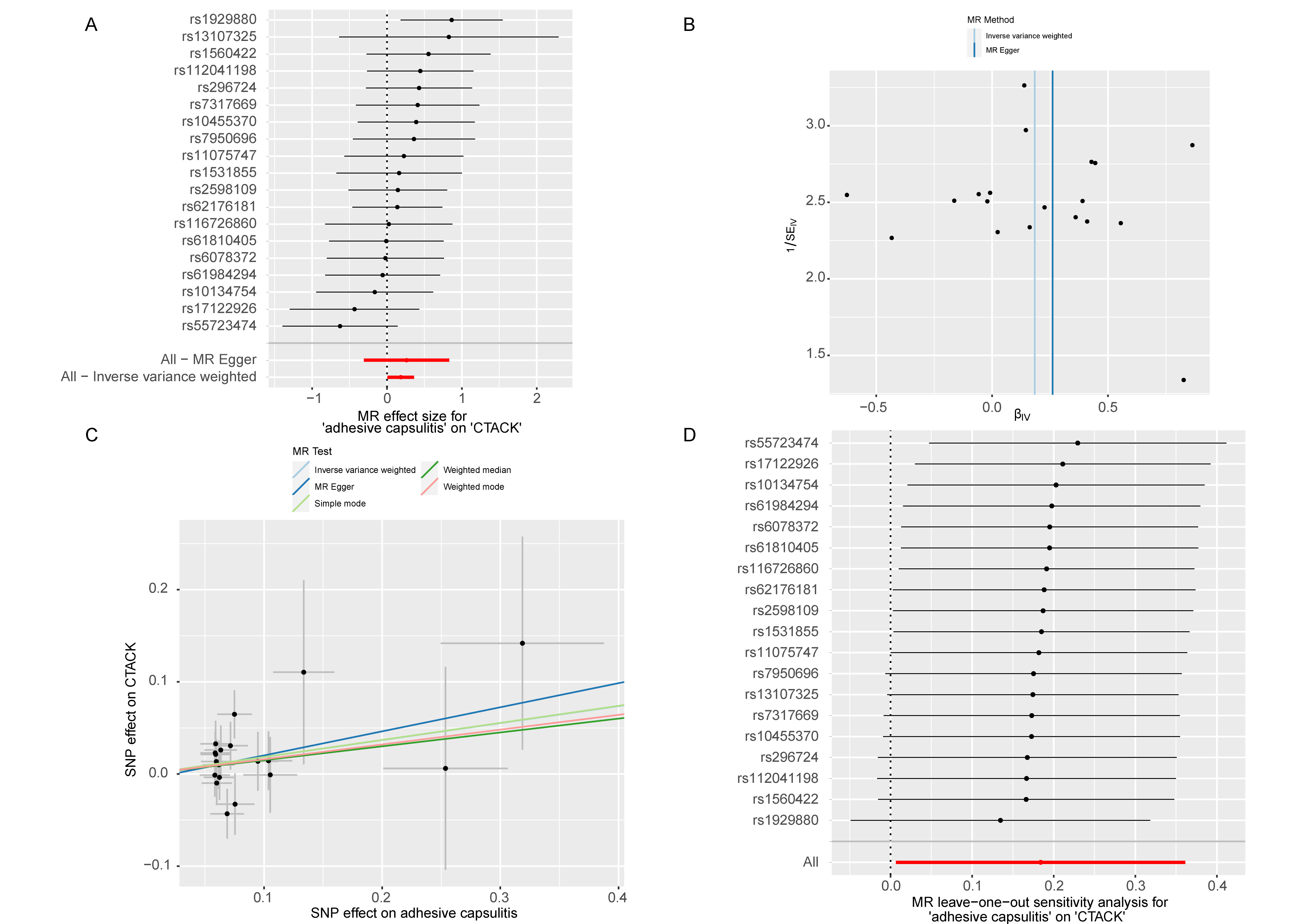


**Supplementary Figure 5** Forest Plot, Funnel Plot, Scatter Plot, and Leave-one-out Analysis (A-D) of adhesive capsulitis on CTACK.


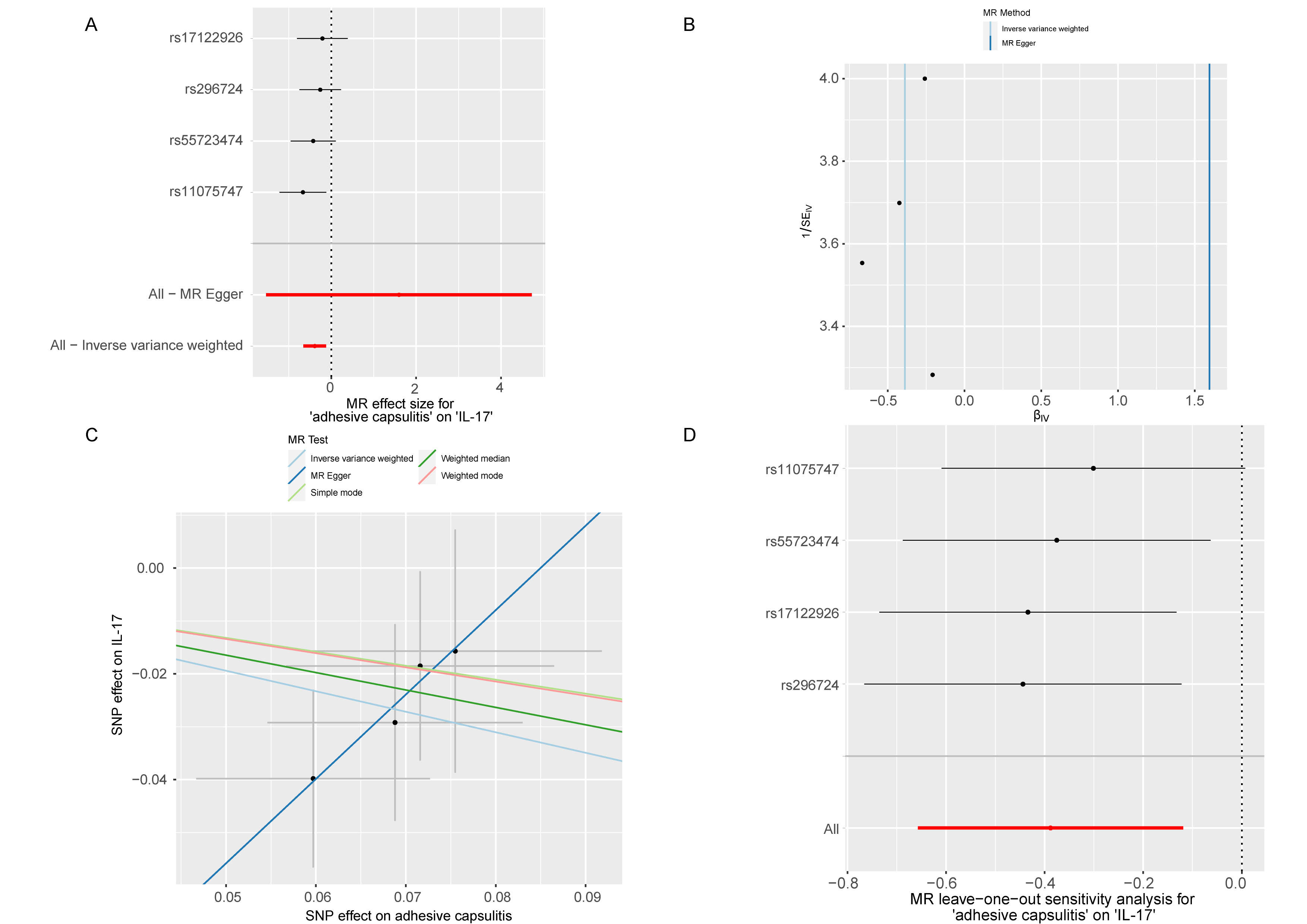


**Supplementary Figure 6** Forest Plot, Funnel Plot, Scatter Plot, and Leave-one-out Analysis (A-D) of IL-17 on CTACK.


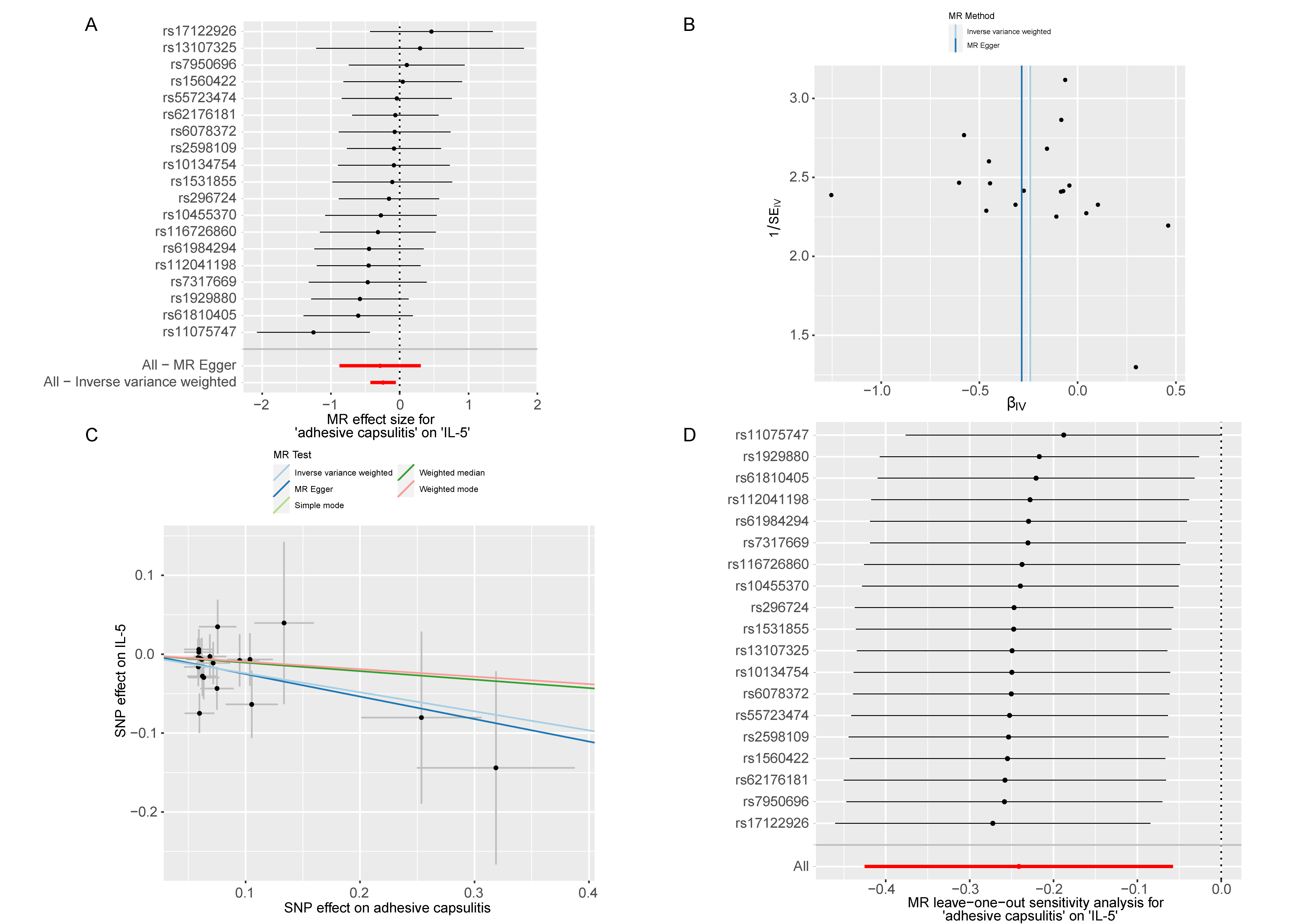


**Supplementary Figure 7** Forest Plot, Funnel Plot, Scatter Plot, and Leave-one-out Analysis (A-D) of IL-5 on CTACK.
